# Putting the treatment of paediatric schistosomiasis into context

**DOI:** 10.1186/s40249-017-0300-8

**Published:** 2017-04-07

**Authors:** Takafira Mduluza, Francisca Mutapi

**Affiliations:** 1 Biochemistry Department, University of Zimbabwe, P.O. Box MP167, Mount Pleasant, Harare, Zimbabwe; 2 School of Laboratory Medicine and Medical Sciences, College of Health Sciences, University of KwaZulu Natal, Durban, South Africa; 3 Institute of Immunology & Infection Research, University of Edinburgh, Ashworth Laboratories, King’s Buildings, Charlotte Auerbach Road, Edinburgh, EH9 3FL UK

**Keywords:** Paediatric schistosomiasis, Praziquantel, Child-size medicine, Target product profile, Drug pipeline

## Abstract

Despite increased international efforts to control schistosomiasis using preventive chemotherapy, several challenges still exist in reaching the target populations. Until recently, preschool-aged children had been excluded from the recommended target population for mass drug administration, i.e. primary school children aged 6–15 years. Our studies and those of others provided the evidence base for the need to treat preschool-aged children that led to recommendations by the World Health Organization to include preschool-aged children in treatment programmes in 2010. The major challenge now lies in the unavailability of a child-size formulation of the appropriate anthelmintic drug, praziquantel.

The currently available formulation of praziquantel presents several problems. First, it is a large tablet, making it difficult for young children and infants to swallow it and thus requires its breaking/crushing to allow for safe uptake. Second, it is bitter so it is often mixed with a sweetener to make it palatable for young children. Third, the current formulation of 600 mg does not allow for flexible dose adjustments for this age group. Thus, there is a need to formulate a child-appropriate praziquantel tablet.

This paper discusses the target product profile for paediatric praziquantel, as well as knowledge gaps pertinent to the successful control of schistosome infection and disease in preschool-aged children.

**Electronic supplementary material:**

The online version of this article (doi:10.1186/s40249-017-0300-8) contains supplementary material, which is available to authorized users.

## Multilingual abstracts

Please see Additional file 1 for translation of the abstract into the six official working languages of the United Nations.

## Background

Schistosomiasis or bilharzia is a water-borne parasitic infection that results in a debilitating chronic disease with extensive morbidity and organ pathology [1–3]. It is endemic in 76 countries worldwide, with about 207 million people infected of which 123 million are children. The majority (88%) of the people infected with schistosomiasis live on the African continent [2, 3].

There are two major types of schistosomiasis disease manifestations: urogenital schistosomiasis (most prevalent in Africa) caused by *Schistosoma haematobium*, and intestinal schistosomiasis, caused by, depending on the tropical region of the world, either *S. intercalatum, S. mansoni, S. japonicum, S. guineensis* or *S. mekongi* [4–6]. Schistosomiasis is implicated in several clinical conditions including bladder cancer leading to death, liver cirrhosis, hydronephrosis, reproductive complications [7], and human immunodeficiency virus (HIV) transmission and fast progression to acquired immune deficiency syndrome (AIDS) in adults [8]. In children, it is associated with poor growth, malnutrition, poor cognitive development, iron-deficiency anaemia and reduced school performance in the high-risk age group (5–15 years) [5, 6].

Encouragingly, several countries in Africa have now embarked on national schistosome control programmes utilising mass drug administration (MDA). The mass treatments are targeting primary school-aged children following treatment regimens recommended by the World Health Organization (WHO) [8]. Currently, the goals for neglected tropical disease control and research are shifting from control to elimination as articulated in the Sustainable Development Goal 3 [9]. For schistosomiasis, the main control strategy is preventive chemotherapy. However, for successful elimination, the approach needs to be strengthened by integrating other control methods such as early diagnosis and coverage of the whole population in endemic areas, as well as integrating these efforts with snail elimination. Global control efforts need to be inclusive of other stakeholders such as primary health centres, family health clinics and primary school health initiatives, as well as other sectors such as agriculture, occupations working in the aquatic environment and, of course, water and sanitation programmes. Inclusive preventative chemotherapy programmes targeting all sectors of the population including out-of-reach communities and children out of school, together with the control of the snail intermediate host and health education, would have a greater and longer-lasting impact than chemotherapy alone. This also means that for elimination to be successful, all animals and human reservoirs of infection must be targeted for treatment, which translates into targeting whole populations in endemic areas.

This review article brings into context the details of global schistosomiasis control that have been overlooked during planning for mass treatment, gaps in the current MDA programmes, the need for a child-size praziquantel tablet, the need for appropriate diagnostics for paediatric schistosomiasis, and the need for complementary control strategies to curtail recontamination of water sources and reinfection.

The clinical assessment of infection and morbidity due to schistosomiasis has mainly been based on haematuria and the presence of eggs in either urine or stool. Detailed confirmation of infection for surveillance purposes, however, has been based on prevalence, infection intensity, clinical presentations and mortality. Introduction of mass treatment has resulted in a sharp decrease of the prevalence as well as the intensity of infection, and the morbidity of the disease has significantly declined. The clinical presentation of acute schistosomiasis has not been utilised appropriately for infection diagnosis, especially in endemic areas where there are several other infections with similar clinical presentations.. In children and infants, the presentations may include fever, rigor, sweating, headache, general muscular pain, gastrointestinal disturbances, enlargement and tenderness of the liver, and eosinophilia [10]. Heavily infected patients are usually very sick with a high fever, which may lead to death. Most subjects with the infection in endemic areas are chronic cases, with the infection reported to have started during the early years of growth and exposure to the infective sources. Most children below the age of five are symptomless, while some are slightly symptomatic and the impact to their general growth is usually not significant. While the preschool age determines the extent of the disease in later age, if not treated in time, the disease may become advanced causing higher morbidity. Other patients may have a latent symptomless infection all their life.

Most of the mortality of schistosomiasis is seen in the advanced stage of the disease. Individuals acquire the infection, usually repeated and heavy, during childhood. However, after schistosomiasis infection control, there is usually successful transmission control, yet individuals can still develop clinical disease. This is likely to occur in young children and individuals with high water contact such as women. Therefore, disease control is still needed in the post-transmission period, in which at-risk populations are monitored. This is a problem in post-mass treatment for schistosomiasis control in the post-transmission period and so surveillance and suitable intervention are needed in areas where transmission is interrupted [10, 11]. In addition, further studies on the pathogenesis of post-transmission schistosomiasis are necessary.

The data from current efforts in different countries will require appropriate management in order to monitor the trends of the programmes and evaluate their efficacy. To analyse the efficacy of schistosome control on a continental scale, a strategy for data collection, management and analysis is required. This may also rely on novel approaches to analyse and interrogate the data, as has recently been highlighted by Walker and colleagues [9]. On the laboratory side, field and laboratory diagnostics with higher sensitivity and specificity during surveillances are required, and for eventual success, there is a need for high uptake of MDA by communities. Current diagnostics need perfecting to be broadly specific and highly sensitive for detecting infections, especially after rounds of preventive chemotherapy treatment efforts through MDA. After rounds of MDA, many areas would experience low infection levels, where the point-of-care circulating cathodic antigen urine cassette test for *S. mansoni* diagnosis may be appropriate for use and there is probably need for improvement of the test for *S. haematobium* diagnosis in the endemic areas. [12]. In such low endemic areas, there is a need to establish new cut-offs/thresholds for diagnosis in regard to low prevalence. In addition, new diagnostic tools to support control programmes leading to and evaluating transmission interruption or even elimination of schistosomiasis need to be evaluated and scaled up in the form of an up-converting phosphor technology-based lateral flow antigen assay [11].

## Paediatric schistosomiasis: infection and morbidity

**Fig. 1:**
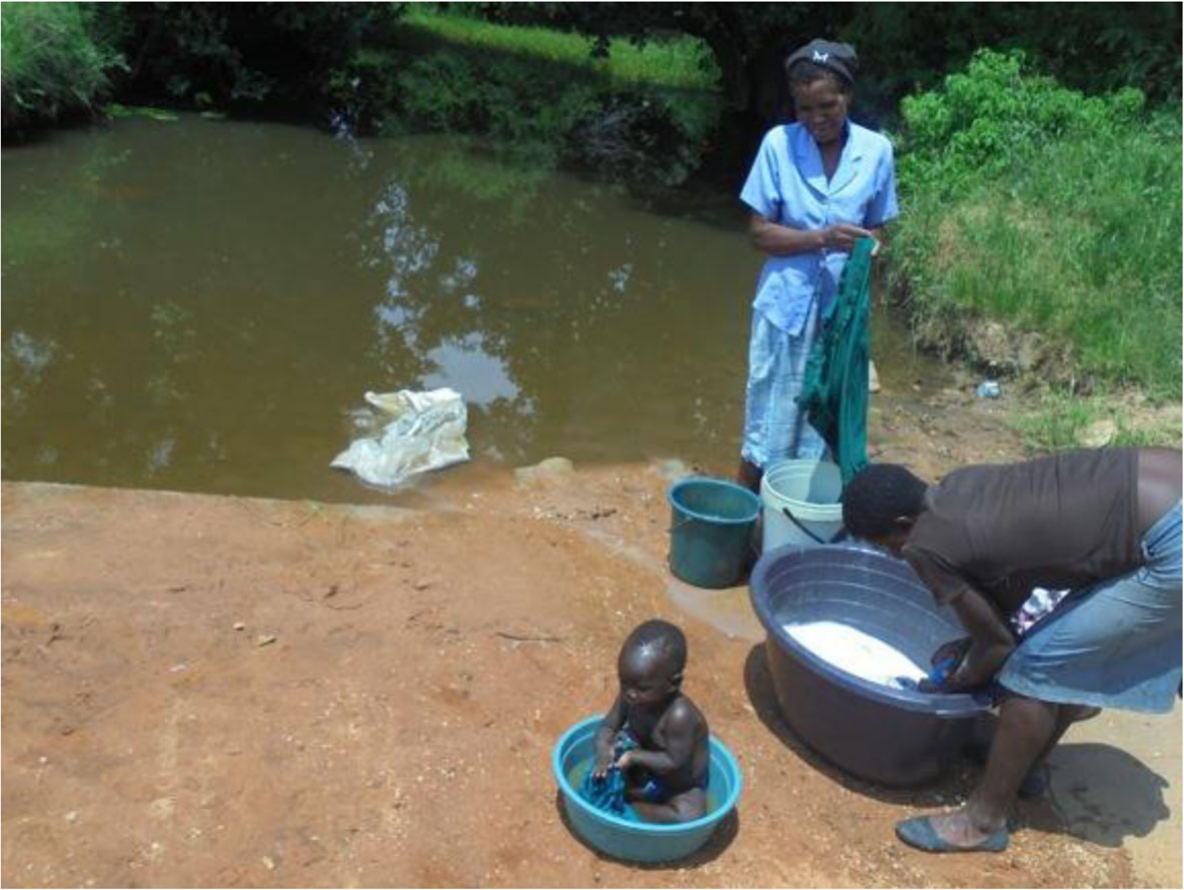
While the mother is busy with laundry chores, the child is placed in a dish with water collected from the river, exposing the child to infection if the water contains cercariae. This was observed to be a common practice in the community where children aged below five years were found to harbour the *S. haematobium* infection

One population exposed to schistosome infection that is still neglected in terms of schistosome treatment and research is the preschool-age group (five years and below). Until recently, preschool-aged children were excluded from schistosome treatment largely due to a lack of evidence on the need for treatment in this group, as well as a lack of age-specific safety and efficacy data. Field studies have shown that young children and infant children can be passively exposed to schistosome infection, for example by being bathed in infective water collected from rivers or playing in infective water while their mothers or caregivers undertake their domestic water-related chores at infested water contact points, thus leading to mothers unknowingly exposing their children to infections (see Fig. 1). As children grow, their exposure patterns change from passive exposure, predominating in young infants as their exposure is linked to that of their careers, to active exposure, as they become more able to frequent contaminated water sources independently with older siblings and friends. Studies by others and us have previously demonstrated that young African children in several countries including Nigeria, Cote d’Ivoire, Kenya, Mali, Uganda and Zimbabwe are infected with schistosomes [13–19]. Furthermore, the limited investigations describing and quantifying morbidity in this age group have shown that the infections in these young children are of clinical significance [20–22], indicating the need to ensure that the preschool-age group is eligible for preventative chemotherapy for schistosomiasis.

Following the recommendations by the WHO in 2010 that this age group requires treatment and the conclusion following several safety and efficacy studies that praziquantel is both safe and efficacious for use in this age group, there has been an uptake to include younger children in schistosome MDA programmes, albeit slowly. In studies involving preschool-aged children in sub-Saharan Africa, including our own in Zimbabwe (for example [11, 16]), the challenge during treatment of infants and younger children has been the lack of an appropriate child-size praziquantel tablet. When treating young children with the currently available commercial praziquantel tablets, there is a need to break them to get the correct dosage, followed by crushing them to avoid chocking, and then finally dissolving or mixing with a sweetener to reduce the bitter taste. While this may be practical for one or a few children, the operation becomes cumbersome when dealing with tens or hundreds of young children and infants, as was the case in our studies, and as would occur to an even larger extent during MDA.

While it is now acknowledged that preschool-aged children are exposed to infection and do get infected with schistosomes, there is still some distance to go in order to define and fully quantify the health impacts attributable to schistosomiasis in this age group. There is a need for more sensitive diagnostics, such as serology, faecal occult blood or micro haematuria to reveal early-stage infections, thereby providing disease diagnostics and treatment before early signs of serious morbidity and pathology. Some reports on *S. mansoni* infections using ultrasonography have highlighted that advanced disease such as chronic fibrosis can be traced back to preschool-age childhood [10, 23].

Paediatric disease case management for clinical schistosomiasis is rare in most endemic areas, since the disease is poorly recognised and rarely considered in younger children before the school-going age. Very few health personnel in rural settings understand the range of schistosomiasis clinical manifestations related to early child health. More detailed studies of exposure patterns are required in this age group to quantify the relative contributions of passive versus active exposure [24–26], as well as to understand the course of pathology during the early stages of infection and child development. The health impact of paediatric schistosomiasis is likely to be greater than currently appreciated. Several studies have demonstrated that children experience the greatest morbidity due to schistosomiasis that manifests in fever and inflammation, which leads to poor health and food intake, resulting in undernutrition, in these settings [27]. Early care for and attention to this age group may provide an opportunity to treat schistosomiasis-associated anaemia, reduce occult blood loss and also reduce inflammatory responses [28]. Schistosome-related inflammation may predispose to poor child growth, as well as decreased efficacy of childhood vaccines [12, 28]. Thus, treatment of schistosomiasis has the dual benefit of treating inflammation due to infection and maximising the efficacy of childhood vaccinations. Early treatment of children in *Schistosoma*-endemic regions may therefore enhance downstream benefits of vaccine programmes, improve educational attainment and cognitive ability, as well as improve overall health outcomes [21, 25].

## Praziquantel target product profile and accessing preschool-aged children for treatment

As outlined earlier, the current existing praziquantel tablet has to be broken into small pieces and sometimes crushed for the under-five-year-old child to take. Studies by others and us have described the limitations of the current tablet formulation for the treatment of preschool-aged children [22, 25, 28], which include tablet size, tablet dose and poor palatability [24–26]. There is a need to improve on the currently available tablet. In addition to being efficacious, a clear target product profile for paediatric praziquantel has been deduced from our studies, which includes, but is not limited to, the following:

1. safe and well tolerated with little to no side effects;
2. small and easy to swallow to avoid chocking;
3. orally disintegrated and can be taken with or without water (in cases of unsafe water supply);
4. improved taste for minimisation of bitterness, easy dose adjustment by number of tablets to avoid having to break tablets; and
5. stability in hot and humid conditions.

To reach all eligible children, the paediatric formulation must also be affordable, preferably costing no more that the current praziquantel tablet, priced at US$ 0.30 per tablet as in most sub-Saharan region.. On the research side, there is a need to optimise treatment by determining the most efficacious dose, as well as the most optimal treatment time and regimen (e.g. frequency of treatment), for these young children. Once we have a paediatric formulation of praziquantel satisfying these requirements, the next aspect of treating younger children is dependent on how to access them.

Primary school children are easily reacheable for helminth control programmes through nearby schools and health centres. There has been discussion [26, 28] on how to best access preschool-aged children, some of whom may not yet be enrolled in Early Childhood Development centres. In sub-Saharan Africa, the Expanded Programme on Immunization for children below five years of age has shown that this age group is easily accessible through the primary health monthly child care programmes, and in our studies in Zimbabwe we access these children through the primary healthcare system [16, 28]. In other countries, Child Health Days also offer a potential opportunity for easily reaching these age groups.

## Praziquantel pharmacokinetics in preschool-aged children

Recent praziquantel pharmacokinetic and pharmacodynamics studies suggest that the efficacy of the current 40 mg/kg body weight, which is extrapolated from studies in adults, may differ for different schistosome species and may also differ for children [21]. For example, praziquantel is currently given at a dose of 40 mg/kg body weight in Africa [19], and 50 mg/kg for adults and 60 mg/kg for children in South America [21]. The calculation of the dose based on weight and the variability of praziquantel absorption relative to food and drink taken simultaneously leads to differences in the pharmacokinetics of praziquantel in children and adults [21, 22, 29, 30]. The relative impact of this on efficacy and safety of any new formulations will therefore need to be investigated. There will be a need for studies investigating the optimal dose for any paediatric formulation of praziquantel, especially if the formulation excludes the inactive isomer responsible for the bitter taste of the currently available praziquantel tablets. Removal of the non-active ingredients may affect the stability of the new formulation, affecting the pharmacokinetics of the drug and the eventual levels and half-life of the bioactive drug in circulation. The recent development of the child-size praziquantel through the Paediatric Praziquantel Consortium is encouraging. Post-human trials assessment of the new formulation for different *Schistosoma* species and using well-designed studies across the affected areas to cater for differences in human host genetics will be important. The evaluation trials of a new paediatric formulation should target the different endemic countries with different species and also different age groups. As the patent is no longer restrictive, more formulations and trials of praziquantel tablets should be supported to encourage local formulations to be developed in endemic countries. Lessons can be learnt from the experiences of South Africa as they forge ahead to produce anti-retroviral drugs locally in 2017 [31].

## Knowledge gap in paediatric praziquantel pharmacogenomics

Pharmacogenomics and pharmacogenetics help us understand the genetics associated with poor response (i.e. the lack of therapeutic response and resistance to treatment of infectious agents) and poor tolerance (including development of adverse drug reactions, ADRs) to drugs in a healthcare setting. Identification of predictors of drug discontinuation remains a major priority in a healthcare setting [32]. Pharmacogenomics has become an important field of science due to the potential to develop personalised medicine. This is relevant in developing countries where most expertise that is needed to deal with the effects of ADRs are not available, as medical prescription practices are based on the theory of one-size-fits-all.

Development of a child-size praziquantel tablet provides an opportunity to investigate genetic impacts on praziquantel pharmacokinetics. In general, there is a growing appreciation that genomic variants can be used to successfully predict individual responses to drug therapy in terms of efficacy and toxicity. The mechanisms revolve around genes responsible for drug metabolism, drug transport and drug targets (i.e. receptors). Genome-wide association studies and whole genome sequencing technologies are likely to identify gene variants that have not previously been considered to play a role in either the disposition or mode of action of a drug, thus these technologies can be used as major resources for novel insights into pathways involved in drug response [31–34]. Very little pharmacokinetics work has been conducted in African populations [35–37]. In this respect, many serious ADRs are only discovered post-licensure of the medications in advanced phases of clinical trials [38, 39].

## Conclusion

The inclusion of preschool-aged children in MDA programmes is essential for the successful and sustained control of schistosomiasis and critical for eventual elimination. It is encouraging that the global community, including the Global Schistosomiasis Alliance and the Paediatric Praziquantel Consortium, are working on providing the tools to facilitate this, the most urgent being a paediatric formulation of praziquantel.

## Additional files

Additional file 1: Multilingual abstracts in the six official working languages of the United Nations. (PDF 333 kb)

## Abbreviations

ADR: Adverse drug reaction
MDA: Mass drug administration
WHO: World Health Organization

## References

[1] Chitsulo LD, Engels D, Montresor A, Savioli L (2000). Global status of schistosomiasis. Acta Trop.

[2] World Health Organization (2002). Prevention and Control of Schistosomiasis and Soil-transmitted Helminthiasis.

[3] World health Organization (2012). Report of a meeting to review the results of studies on the treatment of schistosomiasis in pre-school-age children.

[4] Webster BL, Southgate VR, Littlewood DTL (2006). A revision of the interrelationships of Schistosoma including the recently described *Schistosoma guineensis*. Int J Parasitol.

[5] Gryseels B, Polman K, Clerinx J, Kestens L (2006). Human Schistosomiasis. Lancet.

[6] van der Werf MJ, de Vlas SJ, Brooker S, Looman CW, Nagelkerke NJ, Habbema JD, Engels D (2003). Quantification of clinical morbidity associated with schistosome infection in sub- Saharan Africa. Acta Trop.

[7] World Health Organisation (2002). Prevention and control of schistosomiasis and soil-transmisted helminthiasis. WHO technical Report Series.

[8] Ndhlovu PD, Mduluza T, Kjetland EF, Midzi N, Nyanga L, Gundersen SG, Friis H, Gomo E (2007). Prevalence of urinary schistosomiasis and HIV in females living in a rural community of Zimbabwe: does age matter?. Trans R Soc Trop Med Hyg.

[9] Walker M, Mabud TS, Olliaro PL, Coulibaly JT, King CH, Raso G (2016). New approaches to measuring anthelminthic drug efficacy: parasitological responses of childhood schistosome infections to treatment with praziquantel. Parasit Vectors.

[10] Chen MG (2014). Assessment of morbidity due to Schistosoma japonicum infection in China. Infect Dis Poverty.

[11] Wami WM, Nausch N, Midzi N, Gwisai R, Mduluza T, Woolhouse M (2015). Identifying and evaluating field indicators of urogenital schistosomiasis-related morbidity in preschool-aged children. PLoS Negl Trop Dis.

[12] Colley DG, Bustinduy AL, Secor WE, King CH (2014). Human schistosomiasis. Lancet.

[13] Garba A, Barkiré N, Djibo A, Lamine MS, Sofo B, Gouvras AN (2010). Schistosomiasis in infants and preschool-aged children: Infection in a single *Schistosoma haematobium* and a mixed *S. haematobium- S. mansoni* foci of Niger. Acta Trop.

[14] Mafiana CF, Ekpo UF, Ojo DA (2003). Urinary schistosomiasis in preschool children in settlements around Oyan Reservoir in Ogun State, Nigeria: implications for control. Trop Med Int Health.

[15] Ekpo UF, Laja-Deile A, Oluwole AS, Sam-Wobo SO, Mafiana CF (2010). Urinary schistosomiasis among preschool children in a rural community near Abeokuta. Nigeria Parasit Vectors.

[16] Mutapi F, Rujeni N, Bourke C, Mitchell K, Appleby L, Nausch N, Midzi N, Mduluza T (2011). *Schistosoma haematobium* treatment in 1–5 year old children: safety and efficacy of the antihelminthic drug praziquantel. PLoS Negl Trop Dis.

[17] Sousa-Figueiredo JC, Basanez MG, Mgeni AF, Khamis IS, Rollinson D, Stothard JR (2008). A parasitological survey, in rural Zanzibar, of pre-school children and their mothers for urinary schistosomiasis, soil-transmitted helminthiases and malaria, with observations on the prevalence of anaemia. Ann Trop Med Parasitol.

[18] Uneke JC, Egede MU (2009). Impact of urinary schistosomiasis on nutritional status of school children in south-eastern Nigeria. Internet J Health.

[19] Poole H, Terlouw DJ, Naunje A, Mzembe K, Stanton M, Betson M (2014). Schistosomiasis in pre-school-age children and their mothers in Chikhwawa district Malawi with notes on characterization of schistosomes and snails. Parasit Vectors.

[20] Meurs L, Mbow M, Vereecken K, Menten J, Mboup S, Polman K. Bladder morbidity and hepatic fibrosis in mixed *Schistosoma haematobium* and *S. mansoni* infections: A population-wide study in Northern Senegal. PLoS Negl Trop Dis. 2012;6(9):e1829. doi:10.1371/journal.pntd.0001829. 10.1371/journal.pntd.0001829 PMC3459828 23029589

[21] Olliaro PL, Vaillant MT, Belizario VJ, Lwambo NJ, Ouldabdallahi M, Pieri OS (2011). A multicentre randomized controlled trial of the efficacy and safety of single-dose praziquantel at 40 mg/kg vs. 60 mg/kg for treating intestinal schistosomiasis in the Philippines, Mauritania, Tanzania and Brazil. PLoS Negl Trop Dis.

[22] Stothard JR, Sousa-Figueiredo JC, Betson M, Green HK, Seto EY, Garba A (2011). Closing the praziquantel treatment gap: new steps in epidemiological monitoring and control of schistosomiasis in African infants and preschool-aged children. Parasitology.

[23] Odogwu SE, Ramamurthy NK, Kabatereine NB, Kazibwe F, Tukahebwa E, Webster JP, Fenwick A, Stothard JR (2006). Schistosoma mansoni in infants (aged < 3 years) along the Ugandan shoreline of Lake Victoria. Ann Trop Med Parasitol.

[24] Bustinduy AL, Friedman JF, Kjetland EF (2016). Expanding praziquantel (pzq) access beyond mass drug administration programs: paving a way forward for a pediatric pzq formulation for schistosomiasis. PLoS Negl Trop Dis.

[25] Fenwick A, Savioli L, Engels D, Robert Bergquist N, Todd MH (2003). Drugs for the control of parasitic diseases: current status and development in schistosomiasis. Trends Parasitol.

[26] Sousa-Figueiredo JC, Betson M, Atuhaire A, Arinaitwe M, Navaratnam AM, Kabatereine NB, Bickle Q, Stothard JR (2012). Performance and safety of praziquantel for treatment of intestinal schistosomiasis in infants and preschool children. PLoS Negl Trop Dis.

[27] Malhorta I, Mungai P, Wamachi A, Kioko J, Ouma JH, Kazura JW, King CL (1999). Helminth and Bacillus Calmette-Guerin-induced immunity in children sensitized in utero to filariasis and schistosomiasis. J Immunol.

[28] World Health Oganization (2010). Report of a meeting to review the results of studies on the treatment of schistosomiasis in preschool-age children.

[29] Meyer T, Sekljic H, Fuchs S, Bothe H, Schollmeyer D, Miculka C (2009). Taste, a new incentive to switch to (r)-praziquantel in schistosomiasis treatment. PLoS Negl Trop Dis.

[30] Bustinduy AL, Waterhouse D, de Sousa-Figueiredo JC, Roberts SA, Atuhaire A, Van Dam GJ et al. Population pharmacokinetics and pharmacodynamics of praziquantel in Ugandan children with intestinal schistosomiasis: Higher dosages are required for maximal efficacy. MBio. 2016;7(4). doi: 10.1128/mBio.00227-16. 10.1128/mBio.00227-16 PMC4992966 27507822

[31] Warnich L, Drogemoller BI, Pepper MS, Dandara C, Wright GEB (2011). Pharmacogenomics research in South Africa: Lessons learned and future opportunities in the Rainbow Nation. Curr Pharmacogenomics Person Med.

[32] Bataller R, North KE, Brenner DA (2003). Genetic polymorphisms and the progression of liver fibrosis: a critical appraisal. Hepatology.

[33] Bethony JM, Quinnell RJ (2008). Genetic epidemiology of human schistosomiasis in Brazil. Acta Trop.

[34] Lubomirov R, Colombo S, di Iulio J, Ledergerber B, Martinez R, Cavassini M, Hirschel B (2011). Association of pharmacogenetics markers with premature discontinuation of first line anti-HIV therapy: An observational cohort study. J Infect Dis.

[35] Pang T (2009). Pharmacogenomics and personalized medicine for the developing world - too soon or just-in-time? a personal view from the world health organization. Curr Pharmacogenomics Person Med.

[36] Manolio TA, Collins FS, Cox NJ, Goldstein DB, Hindorff LA, Hunter DJ (2009). Finding the missing heritability of complex diseases. Nature.

[37] Ashley EA, Butte AJ, Wheeler MT, Chen R, Klein TE, Dewey FE (2010). Clinical assessment incorporating a personal genome. Lancet.

[38] Motsinger-Reif A, Jorgenson E, Relling MV, Kroetz DL, Weinshilboum R, Cox NJ, Roden DM (2013). Genome-wide association studies in pharmacogenomics: successes and lessons. Pharmacogenet Genomics.

[39] Wester K, Jönsson AK, Spigset O, Druid H, Hägg S (2008). Incidence of fatal adverse drug reactions: a population based study. Br J Clin Pharmacol.

