# Supplementary material for: Putting the treatment of paediatric schistosomiasis into context
Source: Infect Dis Poverty. 2017 Apr 7;6:85. doi: 10.1186/s40249-017-0300-8 (PMC5384153; doi:10.1186/s40249-017-0300-8)
Supplement: Supplementary file 1 — Multilingual abstracts in the six official working languages of the United Nations. (PDF 333 kb) [file 40249_2017_300_MOESM1_ESM.pdf]

Translation of the abstract into the six official working languages of the United Nations

وضع علاج البلهارسيا في الأطفال في السياق

تاكافيرا مدولوزا، فرانسيسكا موتابي

الملخص

على الرغم من الجهود الدولية المتزايدة لمكافحة مرض البلهارسيا باستخدام العلاج الكيميائي الوقائي مازال هناك العديد من التحديات القائمة في الوصول إلى المجموعات المستهدفة للعلاج وحتى وقت قريب تم استبعاد الأطفال في مرحلة ما قبل الدراسة من الفئات السكانية المستهدفة للعلاج والموصي بها كهدف للعلاج الكيميائي الشامل وتتمثل هذه الفئة في أطفال المدارس الابتدائية الذين تتراوح أعمارهم بين 6-15 سنة وقد أوضحت الدراسات التي قمنا بإجرائها بالإضافة لدراسات الأخرين مدي الحاجة إلى معالجة الأطفال في مرحلة ما قبل المدرسة وأدت هذه الدراسات إلى توصيات منظمة الصحة العالمية بإدراج الأطفال قبل سن المدرسة في برامج العلاج في عام 2010 ولكن التحدي الرئيسي الحالي يكمن في عدم توافر صيغة مناسبة لحجم الأطفال من عقار البرازكوانتيل المضاد للديدان حيث أن الصيغة الحالية المستخدمة في العلاج تمثل العديد من المشاكل أولها كبر حجم الحبوب والذي يجعلها صعبة للبلع من قبل صغار الأطفال والرضع مما يتطلب كسرها او سحقها للسماح بامتصاص آمن لها و ثاني هذه المشاكل هي الحاجة لخلط الحبوب بشيء حلو المذاق للتخلص من مرارتها وجعلها مستساغة للأطفال الصغار اما ثالث هذه المشاكل فهو أن الصيغة الحالية لأقراص ال 600 مليجرام لا تسمح بعمل تعديلات مرنة مناسبة لهذه الفئة العمرية وبالتالي فإن هناك حاجة إلى صياغة أقراص برازكوانتيل ملائمة لعلاج الأطفال وتناقش هذه الورقة العلمية ملف المنتج المستهدف كبرازكوانتيل مناسب للأطفال والفجوات المعرفية المتعلقة بذلك من أجل الحد من عدوي البلهارسيا والمرض في الأطفال قبل سن المدرسة.

Translated from English version into Arabic by Mohamed R. Habib

重视儿科血吸虫病的治疗

Takafira Mduluza, Francisca Mutapi

摘要

尽管国际社会不断努力通过预防性化疗控制血吸虫病，但是如何惠及目标人群仍然存在一些挑战。直到不久前，学龄前儿童仍然被排除在群体性药物化疗推荐的目标人群（如 6-15 岁学龄儿童）外。我们及其他学者的研究为将学龄前儿童纳入化疗提供了证据，从而促使 WHO 在 2010 年推荐将学龄前儿童纳入治疗规划。现在主要的挑战是没有适合儿童剂型的吡喹酮。目前可供选择的吡喹酮剂型存在几个问题。首先，目前的剂型过大，因此不适合儿童和婴儿吞咽，必须掰开或压碎才能够安全服用。其次，目前的剂型较苦，因此需要添加甜味剂儿童才愿意服用。其三，目前的每片 600 mg 的剂型不适合不同年龄组对用量的弹性需求。因此，需要生产一种适合儿童的吡喹酮片剂。本文讨论适合儿童的吡喹酮剂型所应具备的特性、以及在学龄前儿童成功控制血吸虫病尚存在的知识方面差距。

Translated from English version into Chinese by Men-Bao Qian

CHINESE CENTER FOR DISEASE CONTROL AND PREVENTION
NATIONAL INSTITUTE OF PARASITIC DISEASES

Mettre en contexte le traitement de la schistosomiase pédiatrique

Takafira Mduluza, Francisca Mutapi

Résumé

Malgré les efforts internationaux accrus pour lutter contre la schistosomiase à l'aide de la chimiothérapie préventive, plusieurs défis subsistent encore pour atteindre les populations cibles. Jusqu'à récemment, les enfants d'âge préscolaire étaient exclus de la population cible recommandée pour le traitement de masse, c'est-à-dire les enfants d'âge scolaire de 6 à 15 ans. Nos études et celles d'autres auteurs ont fourni des preuves pour la nécessité de traiter les enfants d'âge préscolaire, et ont conduit aux recommandations de l'Organisation mondiale de la santé pour l'inclusion des enfants d'âge préscolaire dans les programmes de traitement en 2010. Le défi majeur réside maintenant dans l'indisponibilité d'une formulation pédiatrique du praziquantel qui est le médicament antihelminthique approprié. La formulation actuellement disponible du praziquantel présente plusieurs problèmes; d'abord le comprimé est gros, faisant qu'il est difficile à avaler pour les jeunes enfants et les nourrissons, et par conséquent, il faut le casser / écraser afin de permettre une absorption sûre. Deuxièmement, il est amer, de sorte qu'il est souvent mélangé avec un produit sucré pour le rendre agréable au gout pour les jeunes enfants. Troisièmement, la formulation actuelle sous forme de comprimés dosés à 600 mg ne permet pas de bien ajuster la dose pour ce groupe d'âge. Ainsi, il est nécessaire de formuler des comprimés de praziquantel appropriés pour les enfants. Ce manuscrit traite du profil de produit cible (TPP) pour le praziquantel pédiatrique ainsi que des lacunes en matière de connaissances pertinentes pour la lutte efficace contre l'infection et la maladie due aux schistosomes chez les enfants d'âge préscolaire.

Translated from English version into French by GARBA DJIRMAY Amadou

Постановка лечения детского шистосомоза в контекст

Takafira Mduluza, Francisca Mutapi

Аннотация

Несмотря на активизацию международных усилий по контролю за шистосомозом с помощью профилактической химиотерапии, все ещё существует ряд трудностей в покрытии целевых групп населения. До недавнего времени дети дошкольного возраста были исключены из рекомендуемых целевых групп населения для массового распределения лекарственных средств, а именно, дети младшего школьного возраста в возрасте 6-15 лет. Наши исследования, а также и другие исследования, создали доказательную базу для понимания необходимости лечения детей дошкольного возраста, что привело к рекомендациям Всемирной организации здравоохранения включить детей дошкольного возраста в программы лечения в 2010 году. В данный момент основная сложность заключается в отсутствии лекарственной формы соответствующего противогельминтного препарата празиквантел, адаптированной для детей. Доступная в настоящее время лекарственная форма празиквантела представляет несколько проблем. Во-первых, большой размер делает её проблемной для приема маленькими детьми и младенцами:

соответственно, чтобы обеспечить безопасный прием, необходимо предварительно разломать или измельчить таблетку. Во-вторых, она горькая, поэтому её часто смешивают с подсластителем, чтобы сделать вкусной для маленьких детей. В-третьих, существующая лекарственная форма в виде таблетки 600 мг не позволяет гибко корректировать дозы для этой возрастной группы. Таким образом, существует потребность в разработке соответствующих детских таблеток празиквантел. Данная работа обсуждает целевой профиль продукта для детского празиквантела, а также пробелы в знаниях, важные для успешного контроля над инфецированием шистосомами и заболеванием у детей дошкольного возраста.

Translated from English version into Russian by Dmitry Esin

Poniendo en contexto el tratamiento de la schistosomiasis pediátrica

Takafira Mduluza， Francisca Mutapi

Resumen

A pesar del aumento de los esfuerzos internacionales para controlar la schistosomiasis mediante la quimioterapia preventiva, todavía existen varios desafíos para llegar a las poblaciones diana. Hasta hace poco, los niños en edad preescolar habían sido excluidos de la población diana recomendada para la Administración de Medicamentos en Masa, es decir, los niños de la escuela primaria, de 6 a 15 años de edad. Tanto nuestros estudios como otros proporcionaron la evidencia básica sobre la necesidad de tratar a los niños en edad preescolar, lo cual llevó a la Organización Mundial de la Salud a recomendar la inclusión de los niños en edad preescolar en programas de tratamiento en 2010. Ahora el principal reto radica en la falta de disponibilidad de una formulación adecuada para la población infantil del fármaco antihelmíntico apropiado, praziquantel. La formulación actualmente disponible de praziquantel presenta varios problemas: primero, es grande, siendo difícil de tragar para la población infantil y por tanto requiere rotura/trituración para permitir su toma de forma segura; en segundo lugar, es amargo por lo que a menudo se mezcla con un edulcorante para que sea de agradable sabor para los niños pequeños; en tercer lugar, la formulación actual en comprimidos de 600 mg no permite ajustes de dosis flexibles para este grupo de edad. Por lo tanto, es necesario formular un comprimido de praziquantel apropiado para niños. Este artículo analiza el perfil del producto diana para el praziquantel pediátrico, así como las lagunas de conocimiento relevantes para el control exitoso de la infección por schistosomas y de la enfermedad en niños preescolares.

Translated from English version into Spanish by Màrius V. Fuentes
